# The C Isoform of *Dictyostelium* Tetraspanins Localizes to the Contractile Vacuole and Contributes to Resistance against Osmotic Stress

**DOI:** 10.1371/journal.pone.0162065

**Published:** 2016-09-06

**Authors:** Tineke Albers, Markus Maniak, Eric Beitz, Julia von Bülow

**Affiliations:** 1 Department of Medicinal and Pharmaceutical Chemistry, Christian-Albrechts-University of Kiel, Kiel, Germany; 2 Department of Cell Biology, University of Kassel, Kassel, Germany; Université de Genève, SWITZERLAND

## Abstract

Tetraspanins (Tsps) are membrane proteins that are widely expressed in eukaryotic organisms. Only recently, Tsps have started to acquire relevance as potential new drug targets as they contribute, via protein-protein interactions, to numerous pathophysiological processes including infectious diseases and cancer. However, due to a high number of isoforms and functional redundancy, knowledge on specific functions of most Tsps is still scarce. We set out to characterize five previously annotated Tsps, TspA-E, from *Dictyostelium discoideum*, a model for studying proteins that have human orthologues. Using reverse transcriptase PCRs, we found mRNAs for *TspA-E* in the multicellular slug stage, whereas vegetative cells expressed only *TspA*, *TspC* and, to a lesser extent, *TspD*. We raised antibodies against TspA, TspC and TspD and detected endogenous TspA, as well as heterologously expressed TspA and TspC by Western blot. *N*-deglycosylation assays and mutational analyses showed glycosylation of TspA and TspC *in vivo*. GFP-tagged Tsps co-localized with the proton pump on the contractile vacuole network. Deletion strains of *TspC* and *TspD* exibited unaltered growth, adhesion, random motility and development. Yet, *tspC*^*−*^ cells showed a defect in coping with hypo-osmotic stress, due to accumulation of contractile vacuoles, but heterologous expression of *TspC* rescued their phenotype. In conclusion, our data fill a gap in *Dictyostelium* research and open up the possibility that Tsps in contractile vacuoles of e.g. *Trypanosoma* may one day constitute a valuable drug target for treating sleeping sickness, one of the most threatening tropical diseases.

## Introduction

**Fig 1:**
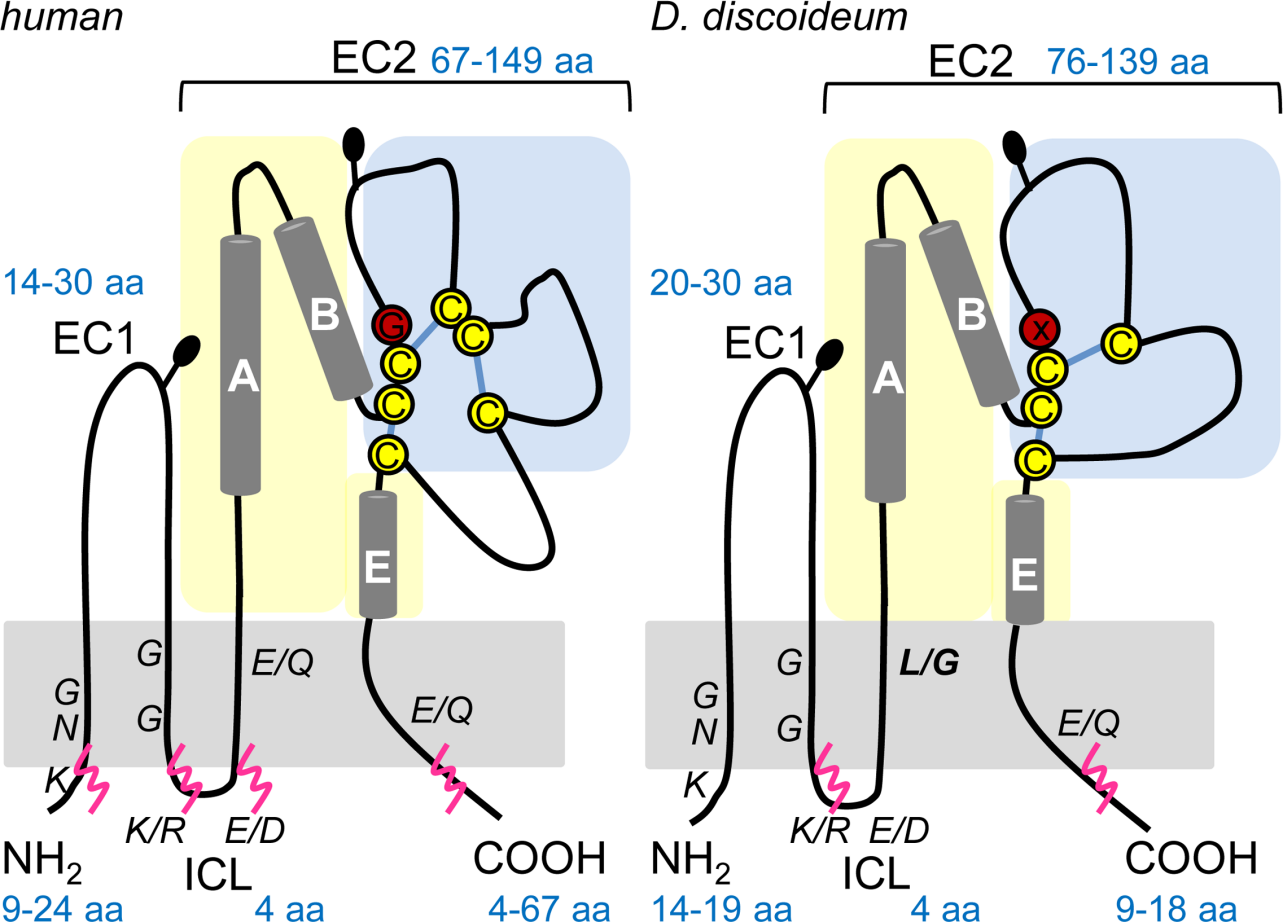
Schematic representation of human and *D*. *discoideum* Tsp topologies. A generic topology of human Tsp is shown on the left (adapted from [33] and [5]), the proposed *Dictyostelium* Tsp topology on the right, as inferred from protein structure predictions and protein sequence alignments. Numbers in blue indicate the range of amino acids (aa) in the N-terminus, the small (EC1) and large (EC2) extracellular loops, the inner cytoplasmic loop (ICL) and the C-terminus. Blue and yellow shadings represent the variable (protein-protein interactions) and conserved domain of EC2, respectively. Cysteines in yellow are 100% conserved, red circles represent the “CCG”-motif that is altered (X) in four of five *D*. *discoideum* Tsps. Conserved residues in the transmembrane regions are indicated in italic letters. Potential disulfide bridges are represented by blue lines. Palmitoylation sites as predicted by CSS Palm 1.0 [58] are marked by pink wavy lines. Black pins show potential *N*-glycosylation sites (N x T/S). Please note that not all Tsps may undergo these modifications to the same extent.

Tsps are a superfamily of integral membrane proteins of 20–30 kDa that were first identified in mammals as cell-specific antigens [1] and later in insects, worms, sponges [2,3], fungi (but not in yeast) [2,4] and plants [5]. To date, 33 distinct Tsps have been found in humans, 37 in *Drosophila melanogaster*, and 20 in *Caenorhabditis elegans* [2,6]. Members of the Tsp family derive their name from four transmembrane domains (TMs). They have cytoplasmic tails at the N- and C-termini, a small extracellular loop (EC1), a small intracellular loop (ICL), a large extracellular loop (EC2) containing a conserved Cys-Cys-Gly (CCG)-motif and two to six additional cysteines (Fig 1, [7]). Mainly localized in the plasma membrane, Tsps form complexes, so-called tetraspanin-enriched microdomains (TEMs), by interacting with a variety of proteins including other Tsps, integrins, growth factor receptors, intracellular signaling molecules and receptor tyrosine kinases [8–12]. Most of these protein-protein interaction sites as well as most monoclonal antibody epitopes map to the extracellular loop EC2. Several lines of evidence indicate that Tsps, respectively Tsps as a part of TEMs, play roles in physiological processes such as cell differentiation, adhesion, motility, cell signaling and sperm-egg fusion [6,9,11] as well as in pathophysiological processes, including cancer metastasis and infections caused by pathogenic organisms [13,14]. Some of these functions have been linked to post-translational modifications of Tsps. Palmitoylation of cytoplasmic, juxtamembrane cysteines is thought to be required for initiating TEM formation [15–17] and *N*-glycosylation has been shown to play important roles in protein interactions, cell adhesion and motility [6,18]. Due to their plasma membrane location with key interacting domains on the extracellular side, Tsps represent tumor markers and potential drug targets that could be addressed by monoclonal antibodies as well as by small molecules [19]. Nearly all types of cells and tissue contain multiple Tsps, often expressed at 30,000–100,000 copies per cell [8,9]. Despite their large number and implication in a broad spectrum of important cellular activities, only a relatively small number of Tsps have been studied in detail [9]. The major obstacles to understanding Tsps are their subtle effects and functional redundancy [2]. Recently, completed genome projects revealed that *Tsp* genes are also found in a few protozoan amoebae such as *D*. *discoideum*, *Trypanosoma brucei* and *Entamoeba histolytica*, albeit with only a few genes per cell type [2,20]. Especially *D*. *discoideum* is a well-established model for studying the cellular role of proteins that have human orthologues [21,22]. *D*. *discoideum* normally lives as a free amoeba but when starved, the cells aggregate to form a multicellular fruiting body. Therefore, this organism provides the opportunity to unravel basic Tsp functions in both, unicellular and multicellular contexts. Yet, surprisingly, physiological and functional data on Tsps in *D*. *discoideum* are completely absent.

In this study, we set out to characterize the predicted Tsps from *D*. *discoideum* [2]. RNA from all five genes was detected in the multicellular slug stage, whereas only three *Tsps* were found to be expressed in vegetative cells. We raised specific antibodies directed against the vegetative Tsps and could detect TspA and TspC by Western blotting. We show by fluorescence microscopy, that the vegetative Tsps co-localize with the V-H^+^ ATPase on contractile vacuoles (CVs). At least for one gene, namely TspC, a gene disruption sensitizes cells for osmotic stress, most likely by delaying the exocytosis of CVs.

## Materials and Methods

*D*. *discoideum cell culture*, *growth and development assays*. *D*. *discoideum* AX2 cells were cultured axenically at 22°C [23]. For growth assays, amoebae in mid-log phase were diluted to an OD_600_ of ~ 0.1 in HL5 medium (Formedium) with 0.5% glucose and agitated at 22°C with 150 rpm. The cell density was monitored by photometric measurements for 96 h. The doubling time was calculated from four independent cultures. To induce development, cells were washed twice in ice-cold Sörensen phosphate buffer (SPB, 15 mM KH_2_PO_4_, 2 mM Na_2_HPO_4_, pH 6.0) and 5 × 10^6^ cells were spread on 1% KK2 agar plates (20 mM KH_2_PO_4_/K_2_HPO_4_, pH 6.8, with 1% agar). Plates were incubated in a moist-chamber at 22°C. Pictures were taken in 4-h intervals for 24 h.

*Preparation of cDNA and PCRs*. For cDNA preparation, 10^7^ vegetative cells in late-logarithmic phase were harvested (1000 × *g*, 4°C, 5 min) and lysed in 1 ml of TRIzol (Invitrogen). For the preparation of slug lysates, development was initiated, slugs were harvested after 16 h and the pellet resuspended in 1 ml of TRIzol. Total RNA was isolated according to the TRIzol protocol. The RNA was isopropanol-precipitated, washed with ethanol 70%, and used for cDNA synthesis (First strand cDNA synthesis kit, Fermentas) using (dT)_18_ primers. PCRs were performed with One Taq DNA polymerase (New England Biolabs) and sequence-specific primers. For details on the PCR primers used see S1 Table (primers 1–10). The PCR programm was as follows: 95°C for 5 min, 30 cyclces of 95°C for 30 s, 55°C for 40 s, and 68°C for 2 min, and 68°C for 10 min.

*Cloning of TspA*, *TspC and TspD and site-directed mutagenesis*. The coding sequences of *TspA*, *TspC* and *TspD* were amplified by PCR from *D*. *discoideum* amoeba cDNA. We used PCR Primers 1–10 (S1 Table). For generation of TspA-His and TspC-His we complemented the respective reverse primer with a 6 bp linker and a sequence coding for a 6x His-tag. The PCR products were ligated into pBluescript II SK(−) for sequencing. DNA point mutations were introduced according to the QuickChange protocol (Stratagene) using Pfu Turbo DNA Polymerase AD (Stratagene). For details on primer sequences see S1 Table (primers 11–14). The PCR programm was as follows: 95°C 30 s, 16 cycles of 95°C 30 s, 55°C for 1 min, and 68°C for 8 min, and 68°C for 20 min. For the generation of GFP fusion constructs, the *Tsp* genes were ligated into pTX-GFP (N-terminal GFP, [24]) using *Bam*H I/ *Xho* I or, after removal of the *Tsp* stop codon, into pDM323 (C-terminal GFP, [25]) using *Bgl* II/*Spe* I.

*Western blot and glycosylation analyses*. *D*. *discoideum* amoebae were harvested in mid-log phase, resuspended in 1 ml of water and lysed by four freeze-thaw cycles at − 80°C and 37°C. Proteins from *D*. *discoideum* cells (30 μg per lane) were separated by SDS-PAGE and transferred to PVDF membranes (Macherey & Nagel). The membranes were incubated with primary antibody. For protein detection we used a commercial polyclonal anti-GFP (Santa Cruz Biotechnology, 1:10,000) and a monoclonal anti-penta-His antibody (Qiagen, 1:2,000) or custom made affinity-purified polyclonal antisera directed against EC2 derived peptides (BioGenes, Berlin, Germany, 1:200). The Western blots were developed with horseradish peroxidase conjugated goat anti-rabbit (Jackson Immuno Research Laboratories, 1:5,000), respectively with conjugated goat anti-mouse (Jackson Immuno Research Laboratories, 1:2,000) antisera using ECL (Amersham Biosciences). For enzymatic deglycosylation analysis, a membrane protein fraction was collected by centrifugation of debris-free amoebae lysates (100,000 × *g*, 4°C, 45 min). *N*-glycosylation of TspA and TspC was assayed by incubation with 1,000 units of *N*-glycosidase F (New England Biolabs) for 60 min at 37°C.

*Expression and localization of Tsp-GFP fusion constructs*. 7 × 10^6^ *D*. *discoideum* amoebae were harvested (2,000 × *g*, 10 min, 4°C), washed twice with H50 buffer (20 mM HEPES, 50 mM KCl, 10 mM NaCl, 1 mM MgSO_4_, 5 mM NaHCO_3_ and 1 mM NaH_2_PO_4_, pH 7.0) and incubated for 5 min in 700 μl of H50 with 4–10 μg of plasmid DNA. Electroporation was done in 0.4 mm electroporation cuvettes with two pulses (5 s delay) of 1.2 kV, 50 μF and a time constant of 1–2 ms using a Gene Pulser II (Bio-Rad). After addition of axenic HL5 medium (Formedium) with 0.5% glucose, cells were incubated at 22°C for 24 h before adding 10 μg ml^-1^ G418 for antibiotic selection. Fluorescence imaging was done by confocal microscopy (Leica TCS-SP, 100x NA1.4 objective). Tsp-GFP expressing amoebae were fixed with 2% paraformaldehyde and 15% picric acid in 20 mM PIPES buffer pH 6.0 (Sigma). Cells were washed with PIPES and Phosphate-buffered saline (PBS, 137 mM NaCl, 2.7 mM KCl, 8.1 mM Na_2_HPO_4_ and 1.5 mM KH_2_PO_4_, pH 7.4) with 100 mM glycine and incubated for 10 min in 70% ethanol. CVs were stained as described in [26] with a monoclonal antibody 221-35-2 directed against a subunit of the vacuolar-H^+^ ATPase [27,28], or protein disulfide isomerase 221-135-1 [29] (undiluted culture supernatant) and with a second polyclonal goat-anti mouse Cy3 antibody (Dianova; 1:1,000). GFP fluorescence was excited with an argon ion laser at 488 nm and Cy3 fluorescence with a He/Ne laser at 543 nm.

*Generation of tspC*^*−*^ *and tspD*^*−*^ *strains*. Gene deletion was done as described previously [30], using electroporation with a blasticidin S resistance cassette flanked by two homologous fragments of the *TspC* or *TspD* gene for homologous recombination. Briefly, 5´ and 3´ fragments were amplified from AX2 genomic DNA using specific forward and reverse primers (for details on the PCR primers see S1 Table, primers 15–22). The PCR programm was as follows: 95°C for 5 min, 30 cycles of 95°C for 30 s, 53–56°C for 40 s, and 68°C for 2 min, and 68°C for 10 min.

The PCR products were cloned into pLPBLP via the *Sal* I/*Hin*D III (5´ fragment) and *Pst* I/*Bam*H I sites (3´ fragment). The construct was excised with *Sal* I/*Bam*H I, purified and electroporated into AX2 cells. Transformants were selected for seven days in HL5 containing 10 μg ml^-1^ blasticidin S. Individual colonies were picked with a pipette, transferred into 24-well plates and screened by PCR. For details on the primers used see S1 Table (primers 23–34). The PCR programm was as follows: 95°C for 5 min, 30 cylces of 95°C for 30 s, 50–54°C for 40 s, and 68°C for 2 min, and 68°C for 10 min.

*Adhesion and random motility assays*. Cell-substrate adhesion was performed as described by Garcia *et al*. [31] with slight modifications. A suspension of 1 × 10^6^ cells in 1 ml of SPB was placed in a 125 ml glass culture flask and put on a gyratory shaker for 10 min at 120 rpm. The cells were allowed to adhere to the glass substrate for 2 h. The flask was then gently agitated for 3 min at 60 rpm. The supernatant was collected and the number of cells was counted with a hemocytometer. Random motility was monitored by time-lapse imaging of 7.9 × 10^4^ growth-phase cells per 24-well (Sarstedt, #83.1836) in SPB using an ImageXpressMicro High Content Screening System (Molecular Devices). In each experiment, the x/y positions of 50–100 cells were determined in 15 s intervals for 15 min. Cell motility was analyzed using ImageJ Manual Tracking and Chemotaxis software. Experiments were performed with two independent knockout clones for each *Tsp*.

*Osmoregulation assays*. Exponentially growing cells were placed in a well of a 96 well plate (Greiner bio-one, #655090) and left to adhere for at least 30 min. Pictures were taken with an ImageXpressMicro High Content Screening System (Molecular Devices) with a 100x objective in HL5 medium with 0.5% glucose (0 min). After exchanging the media with distilled water, images were captured at 5, 10, 20, 30 and 40 min. Circularity was measured offline for each cell with ImageJ. Each data point is the mean of 75–116 cells. Error bars represent S.E.M.. Experiments were performed with two independent knockout clones for each Tsp. Chambers for *in vivo* microscopy of contractile vacuoles were made from four fused silicon rings (Flexiperm, Sarstedt) pressed on a 50 x 50 mm square custom-made glass coverslip (Hecht) and maintained in a moist chamber. Cells from shaking culture were seeded into the wells and allowed to adhere for at least 60 min in growth medium. After the medium was withdrawn, cells were rinsed once with SPB and immediately imaged for up to 30 min in fresh SPB containing 1 μg ml^-1^ of the styryl dye FM 4–64 (Invitrogen) using argon ion laser excitation (488 nm) and recording emission in a window from 600 to 800 nm on a Leica TCS-SP confocal microscope.

## Results

### Cloning and sequence analysis of *D*. *discoideum* Tsps

**Fig 2:**
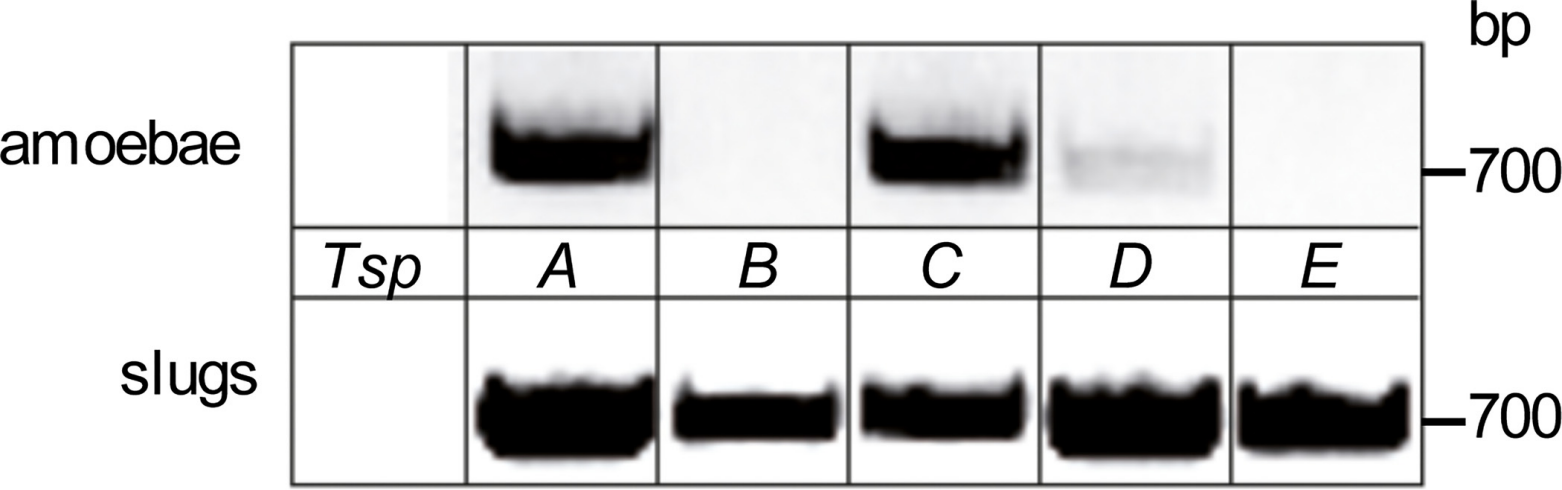
Expression of *TspA-E* in *D*. *discoideum*. Total RNA was isolated from growth phase cells (amoebae) and from cells after 16 h of development (slugs), cDNA was prepared and PCR reactions were performed with sequence-specific primers (S1 Table). PCR products had the predicted sizes of approximately 700 bp.

BLAST searches in the genome sequence of *D*. *discoideum* yielded five novel putative *Tsp* genes that had been annotated by dictybase as *TspA-E* (Table 1, [2], dictybase.org). *TspA-D* are located on chromosome 1, *TspE* on chromosome 5. *TspA-E* consist of ~700 bp with an A + T content of ~ 70%. The flanking non-coding regions have an A + T content of more than 85%, which is typical for intergenic regions in the *D*. *discoideum* genome [32]. The genes of *TspA-E* have one to three introns. We were able to amplify the ORFs of *TspA*, *TspC* and *TspD* from cDNA of *D*. *discoideum* AX2 vegetative amoebae (Fig 2). We also managed to amplify the ORFs of all five *Tsps* from AX2 slugs (Fig 2), indicating that the genes are transcribed and do not represent pseudogenes. All PCR products had the predicted size of approximately 700 bp. Expressed sequence tag data (EST) and expression time courses from dictyExpress (dictybase.org) provide further support for the gene transcription pattern we describe here (Table 1). The three Tsps from vegetative amoebae, TspA, TspC and TspD, were used for further studies. The DNA sequences obtained experimentally were identical to the database entry. A topology prediction on the basis of a protein sequence alignment shows that *D*. *discoideum* TspA-E contain four TMs, a short extracellular loop EC1 (20–30 aa), a very short intracellular loop (ICL, 4 aa) and a large extracellular loop EC2 (76–139 aa), flanked by relative short N- (14–19 aa) and C-terminal (9–18 aa) tails (Fig 1). The EC2 is divided into a constant region (light yellow shading) containing α-helices A, B and E, that is important for homodimerization and a variable region (blue shading), that represents putative protein-protein interaction sites [33]. TspE contains the “CCG”-motif present in all human Tsp EC2s, while the other four Tsps show a modified “CCK/Y/C” motif, which is more similar to some variants present in the plant lineage [5]. *Dictyostelium* Tsps contain the typical four cysteines (yellow circles) that form intramolecular disulfide bonds crucial for correct folding and protein-protein interaction of metazoan EC2s [33]. We also identified multiple consensus sites for secondary protein modifications of *Dictyostelium* Tsps: two potential palmitoylation sites adjacent to TM2 and TM4 (pink wavy lines) and potential *N*-glycosylation sites (black pins) in EC1 and EC2. Most amino acids indicated as important by structural and mutational analysis of the mammalian proteins [34,35] are also present in *Dictyostelium* Tsps (Fig 1). Specifically, this includes conserved polar (Asn/Glu/Gln) and Gly residues in the TMs (indicated in italic letters). In TM3, *Dictyostelium* Tsps have a Leu/Gly where human Tsps have conserved Glu/Gln. However, mutation of Glu103 in TM3 of human CD9 to Ala had no effect on cell surface expression of the protein and its dimerization [35], hinting at a tolerable amino acid exchange.

**Table 1:** Overview of Tsps in *D*. *discoideum*.

Name	Dictybase Gene ID	Predicted protein size (kDa)	“CCG”-motif	Expressed sequence tags (ESTs)	Introns	Chromo-some
TspA	DDB_G0269110	25.5	CCK	5 vegetative, 11 slug	2	1
TspB	DDB_G0269872	25.5	CCK	2 aggregation, 7 slug	2	1
TspC	DDB_G0270986	26.3	CCC	2 slug	3	1
TspD	DDB_G0270682	25.4	CCC	4 slug	2	1
TspE	DDB_G0291177	26.5	CCG	0	1	5

### Expression and glycosylation of Tsps from *Dictyostelium* amoebae

**Fig 3:**
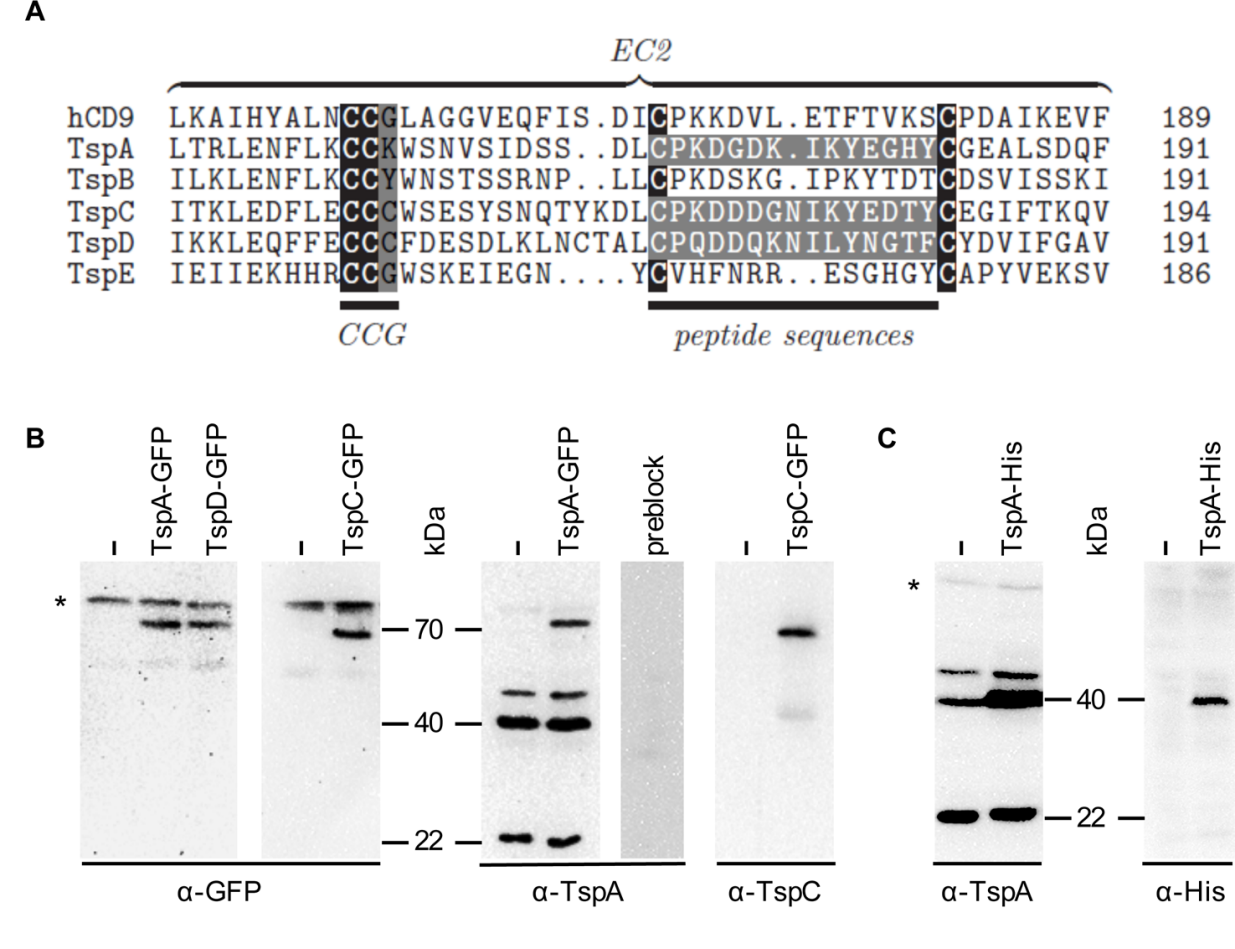
Generation of TspA, TspC and TspD antibodies, Western blots of Tsp-GFP fusion proteins and specification of the TspA antibody. (A) Partial sequence alignment of *D*. *discoideum* TspA-E and human CD9 (the plot was generated using TEXshade [59]). Peptide sequences that were used for antibody production and conserved Cys residues are labeled in white. The conserved “CCG”-motif is indicated. (B) Western blots of cell lysates (30 μg protein per lane) of *D*. *discoideum* vegetative cells transformed with the control plasmid pDM323 (−) or *Tsp-GFP* using a polyclonal α-GFP antibody as well as the affinity purified antibodies α-TspA and α-TspC. The specificity of the anti-TspA antiserum (preblock) was tested by a 1 h preincubation with 1 μg ml^-1^ of the immunizing peptide corresponding to a 10-fold excess. (C) Western blot of cell lysates (88 μg protein per lane) of *D*. *discoideum* vegetative cells (−) and of cells transformed with *TspA-His* using the affinity purified α-TspA antibody as well as a monoclonal α-His antibody. * = unspecific band.

To test for *in vivo* expression by Western blot analysis, we raised antibodies against TspA, TspC and TspD. We used peptide sequences derived from EC2 for antibody production (Fig 3A), as the respective N- and C-terminal tails are too short (Fig 1) and most useful Tsp antibodies have been generated against the EC2 [19]. To test the three affinity-purified antibodies, we generated Tsp proteins fused to green fluorescent protein (GFP) in *D*. *discoideum* vegetative cells. We detected two bands in our Western blots for each Tsp-GFP fusion protein using a polyclonal α-GFP antibody (Fig 3B). We found an 80 kDa band, representing an unspecific signal (Fig 3B, *) that was also detected in cells transformed with a control plasmid as well as a specific band of 70 kDa (Fig 3B). This is a higher mass than the theoretically expected 52 kDa, hinting at potential post-translational modifications, a common feature of Tsps. We used the same cell lysates to test the three affinity-purified antibodies. The TspD antibody depicted no signals. The affinity purified TspA antibody detected the 70 kDa band of TspA-GFP and three additional bands of 22, 40 and 44 kDa that might represent endogenous TspA (Fig 3B). For example, the molecular weight of human CD63 has been observed to be 32, 35 and 50 kDa with *N*-linked glycosylation in Western blot experiments, although the predicted molecular weight of CD63 is 25 kDa [36]. We addressed the specifity of the TspA antibody by preincubating it with the immunization peptide and found that all bands disappear (Fig 3B, preblock). Transformation of *D*. *discoideum* vegetative cells with TspA-His lead to an increase in the intensity of the 40 kDa band compared to the signal from untransfected cells (Fig 3C). The same band was also detected by an α-His antibody (Fig 3C, right). These results strongly suggest that at least the 40 kDa signal represents endogenous TspA.

**Fig 4:**
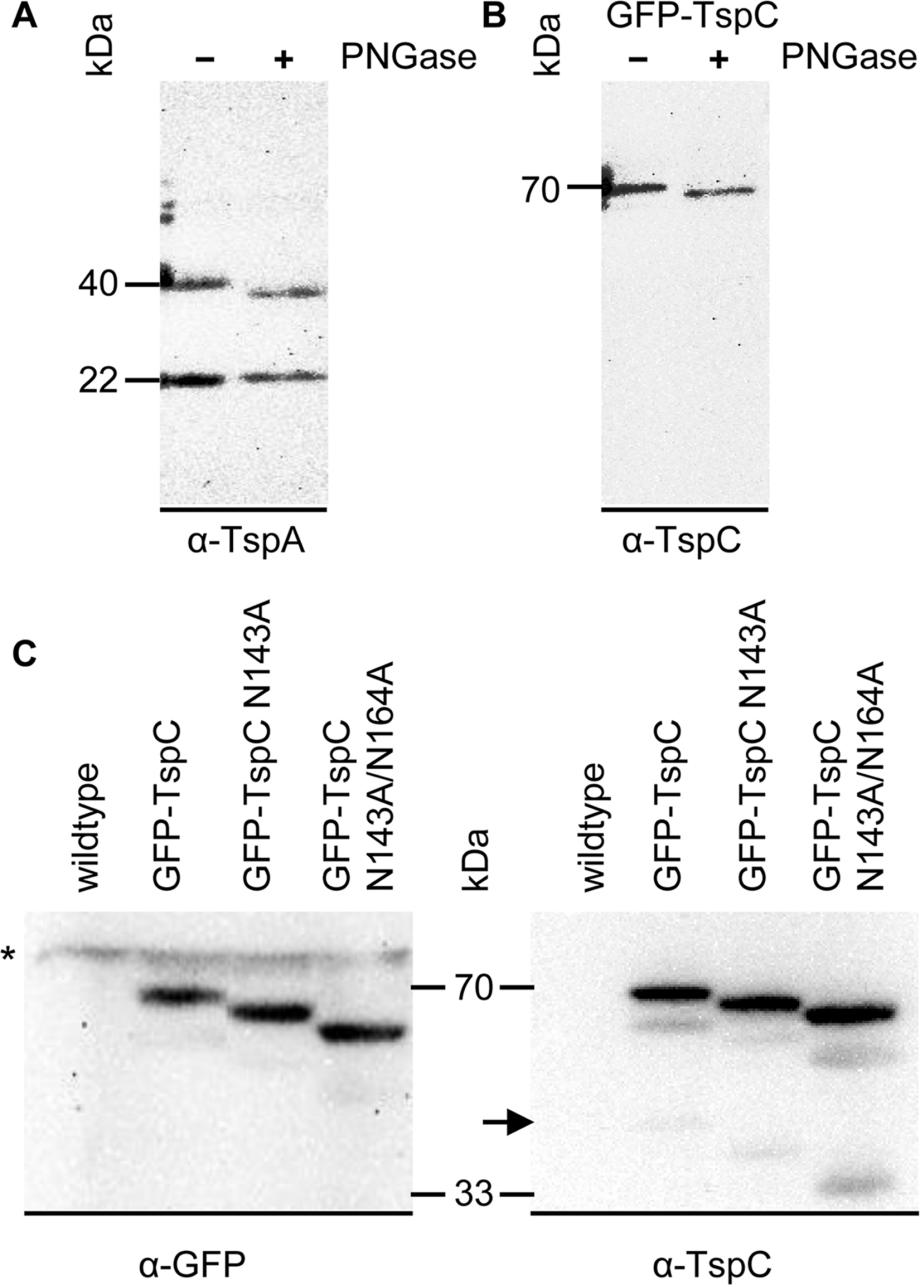
Glycosylation of TspA and TspC. PNGase F was used for enzymatic *N*-deglycosylation of (A) native TspA and (B) heterologously expressed GFP-TspC of *D*. *discoideum* membrane fractions. The shift in molecular weight of the 40 kDa band for TspA or the 70 kDa for GFP-TspC, respectively, after PNGase treatment is shown by Western blot and hints at *in vivo* glycosylation of both proteins. (C) The Western blot shows cell lysates of GFP-TspC and the glycosylation deficient mutants N143A and N143A/N164A. A shift in molecular weight of the 70 kDa band for each mutation hints at glycosylation at both sites. A degradation product of GFP-TspC is also detected by the α-TspC antibody and shows a gradual shift in molecular weight that leads to a 33 kDa band of deglycosylated TspC.

The α-TspC antibody detected the TspC-GFP fusion protein of 70 kDa but, surprisingly, not the endogenous TspC (Fig 3B). A weak signal at 40 kDa was only found in TspC-GFP expressing cells but not in wildtype cells, most likely representing a degradation product. We saw no difference in antibody recognition for N- and C-terminal GFP fusion (Fig 3B and S1 Fig and Fig 4B).

As we found two potential *N*-glycosylation motifs in the TspC-EC2 (Asn143 and Asn164) that lie in close proximity to the antibody recognition site Cys171-Tyr185 (Fig 1), we figured that the difference in antibody recognition may be due to a difference in glycosylation of endogenous and GFP-TspC. To test this hypothesis and also check for *in vivo* glycosylation of TspA, we used an enzymatic approach. Incubation with *N*-glycosidase F (PNGase) shifted the molecular weight of the 40 kDa TspA band (Fig 4A) and the 70 kDa GFP-TspC band (Fig 4B), hinting at *N*-glycosylation of both Tsps. For TspC we proceeded using a mutational approach. We expressed GFP-TspC mutants that lack one (N143A) or both (N143A, N164A) asparagine residues and found a stepwise molecular weight shift of the 70 kDa band, indicating that both positions are glycosylated *in vivo* (Fig 4C). Even lane loading is seen by the unspecific signals (*) throughout all lanes (Fig 4C, left). Using the α-TspC antibody we also obtained a weak signal at 40 kDa for the GFP-TspC degradation product that gained intensity and shifted to lower molecular weight in the respective glycosylation-deficient mutants (Fig 4C, arrow). This observation may hint at the fact that glycosylation in the neighborhood of the epitope interferes with antibody recognition.

### TspA, TspC and TspD co-localizate with V-H^+^ ATPase

**Fig 5:**
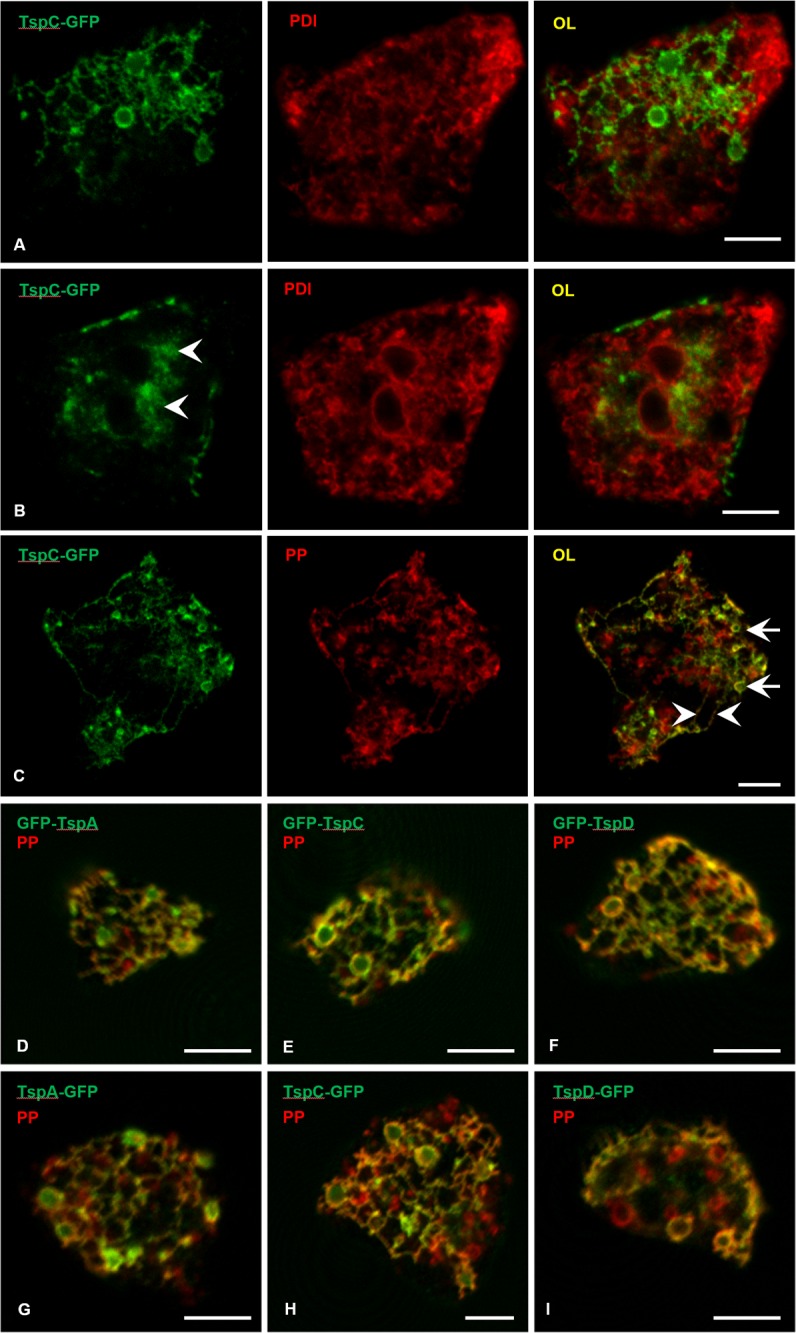
Tsp-GFP fusion constructs co-localize with the proton pump on contractile vacuoles. (A) Fixed amoebae expressing TspC-GFP show a delicate green network with occasional bladders at the bottom of the cell that does not co-localize with a marker of the ER (PDI, red) in indirect immunofluorescence. OL = overlay. (B) In a more medial plane of the same cell, the GFP signal is found within the plasma membrane in addition to having a cloudy appearance (arrowheads). This is indicative of the Golgi-apparatus lying close to the ER-rings that represent the nuclear envelopes. (C) Using the V-H^+^ ATPase (or proton pump, PP, red) as a stain for the CV, the cisternae (arrows) and tubular elements (arrowheads) strongly overlap with TspC-GFP in the overlay (OL). Some vesicular structures only labeled by the V-H^+^ ATPase antibody may be components of the endolysosomal system that is known to be populated by the V-H^+^ ATPases [60]. N-terminal (D–F) as well as C-terminal (G–H) GFP fusions to TspA, TspC and TspD proteins all localize to bladders and tubules of the CV system (merge yellow) counterstained as in (C). Scale bars = 5 μm.

Next, we set out to characterize the subcellular localization of the Tsps from *D*. *discoideum* vegetative amoebae. Our affinity-purified antibodies did not recognize endogenous TspC and TspD and produced several bands in case of TspA. This rendered our antisera unsuitable for immunolocalization. As an alternative, we used N- and C-terminal GFP fusion constructs for the experiments. Unlike the plasma membrane localization typically seen for mammalian Tsps, we found a mainly intracellular localization of the GFP fusion proteins in *D*. *discoideum* amoebae (Fig 5). Close to the surface of the cell that adheres to the coverslip, tagged Tsps accumulated on large vacuoles and reticular structures that radiate from them (Fig 5A, 5C to 5I). This network was distinct from the endoplasmic reticulum (ER), because it did not coincide with the marker protein disulfide isomerase (PDI, Fig 5A and 5B), but instead strongly overlapped with the vacuolar proton pump (PP, Fig 5C to 5I), a well-known marker of the contractile vacuole (CV) system [37]. Because N-terminally (Fig 5D to 5F) and C-terminally (Fig 5G to 5I) GFP-fused Tsp-proteins showed the same localization pattern, we are confident that all the endogenous proteins represent true constituents of the CVs of *D*. *discoideum*. Farther away from the adherent suface, Tsps were seen in patches within the plasma membrane, indicative of exocytosed CVs, and in clouds of vesicles in the vicinity of the cell nuclei (Fig 5B) most likely representing the Golgi apparatus, where the Tsps are thought to receive their glycosylation.

### *TspC*^*−*^ mutant cells have defects in osmoregulation

**Fig 6:**
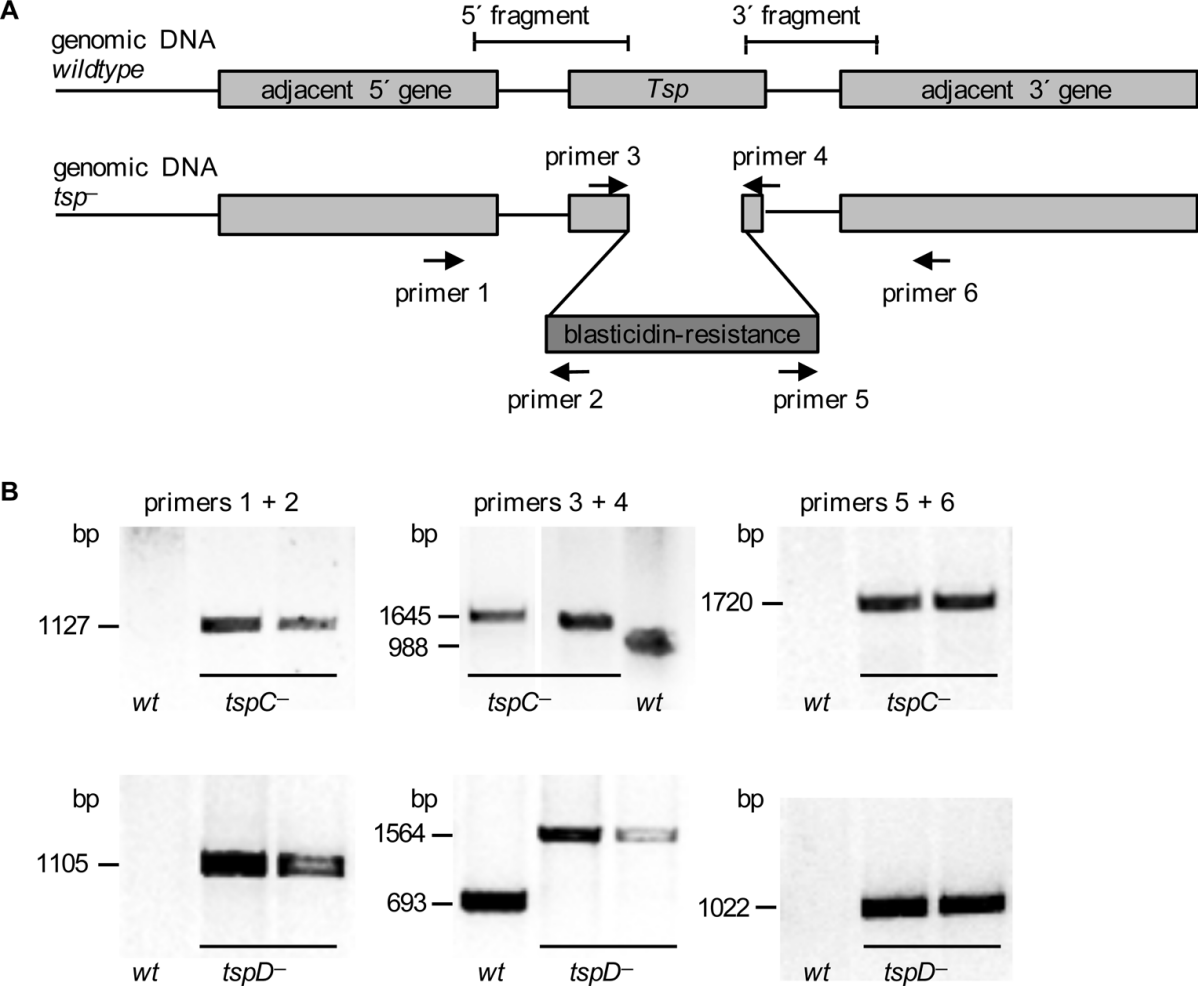
Disruption of the *TspC* and *TspD* genes. (A) Genomic 5´ and 3´ fragments of *TspC* and *TspD* were amplified by PCR, ligated into a blasticidin-resistance cassette and each construct was transferred into AX2 wildtype cells by electroporation. The adjacent genes of *TspC* (*lipocalin*, DDB_G0269882; *pseudogene samkB*, DDB_G0270988) and *TspD* (*TspA*; DDB_G0269884) are shown. (B) For each gene, two independent blasticidin resistant clones were compared with the wildtype (wt) for correct genomic 5´ integration (primers 1/2), insertion of the blasticidin-resistance gene (primers 3/4), and 3´ integration (primers 5/6).

**Fig 7:**
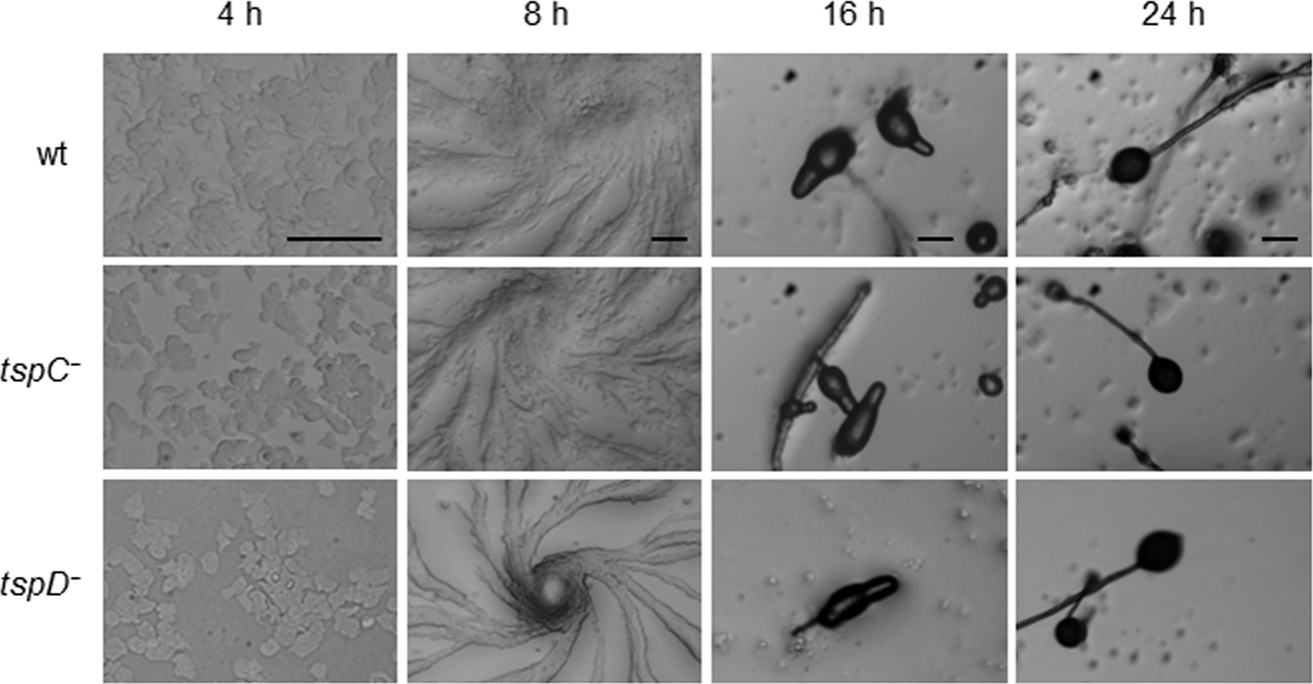
Twenty-four hour development. Cells were starved on KK2 agar plates and incubated at 22°C in a moist chamber. Pictures were taken at the indicated time points illustrating aggregation (4 h), streaming (8 h), culmination (16 h) and spore-head formation (24 h). Development of *tspC*^*−*^, as well as of *tspD*^*−*^ mutant was unaltered compared to wildtype cells. Scale bars = 100 μm.

**Fig 8:**
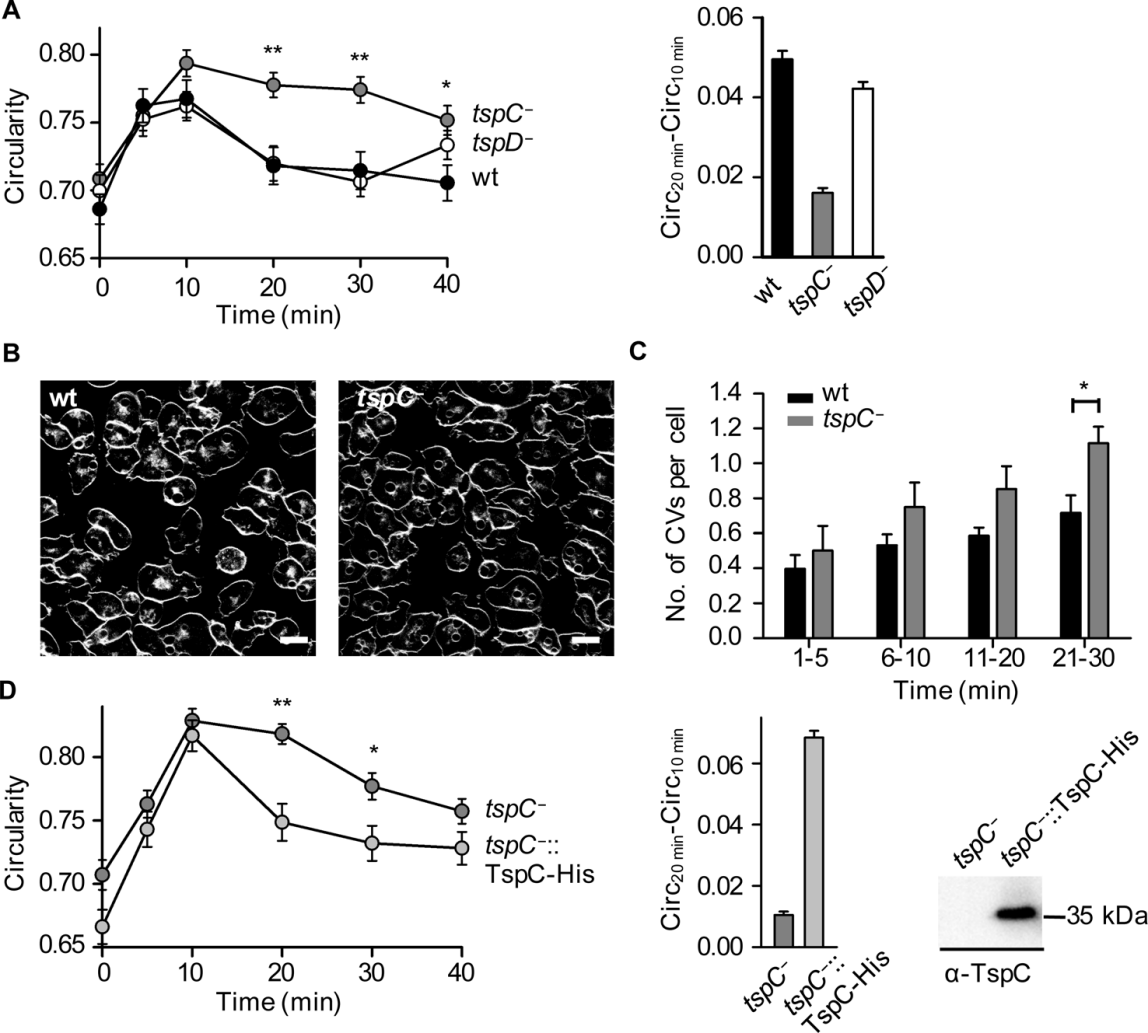
Lack of TspC renders cells osmosensitive. (A) Time course of cells increasing in circularity and their recovery. Wildtype (black circles) and *tspD*^*−*^ (white circles) cells in distilled water round up for 10 min but regain their normal shape by 20 min. *TspC*^*−*^ cells (grey circles) recover significantly slower but show a tendency to become amoeboid after 40 min (** = *P* = 0.001 for 20 and 30 min, * = *P* < 0.05 for 40 min, evaluated with one-way ANOVA with a Bonferroni post-hoc test). Error bars represent S.E.M. and results are means of two independent experiments with a total number of 75–116 cells analysed per strain. The initial recovery rate (right picture) represents the Δcirculartiy between 20 and 10 min. Error bars represent S.E.M.. (B) Confocal sections through living wildtype (wt) and mutant (*tspC*^*−*^) cells after 16 min of incubation with FM4-64 to stain the plasma membrane and the CVs. The size bar is 10 μm. (C) Images such as the ones shown in (B) were grouped into time-frames of 5 or 10 min as indicated and vacuoles were counted for 500 to 600 cells in each time-frame. The average number of CVs per cells is displayed, wildtype (wt) as black and mutant (*tspC*^*−*^) cells as grey bars, error bars represent S.E.M. (* = unpaired t-test, *P* < 0.05). (D) Rescue experiment for the *tspC*^−^ phenotype. Time course of cell increase in circularity and recovery. *TspC*^−^ (grey circles) and *tspC*^−^ cells expressing *TspC-His* (light grey circles, *tspC*^−^::TspC-His) in distilled water round up for 10 min but only *TspC*^−^::TspC-His cells regain their normal shape by 20 min. Again, *TspC*^*−*^ cells exhibit a significant disability to recover before 40 min (** = unpaired t-test for 20 min *P* < 0.001, * = *P* < 0.05 for 30 min). Expressing *TspC-His* can therefore compensate for the osmoregulation defect of *tspC*^−^. Error bars represent S.E.M. and results are means of two independent experiments with a total number of 53–128 cells analysed per strain. The initial recovery rate (middle) represents the Δcirculartiy between 20 and 10 min. Error bars represent S.E.M.. Western blot of *TspC*^−^::TspC-His cells with a 35 kDa band detected with an α-TspC antibody (right).

Because the localization of GFP-tagged Tsps to the CV suggests that they might play a role in the function of this organelle, we generated *D*. *discoideum* strains by disrupting the respective genes (Fig 6A). In case of *TspA*, we were not able to generate viable knockout cells from several independent transformations. At present, we cannot say if this is due to an unsuitable construct, or if a lack of *TspA* affects viability of *D*. *discoideum* amoebae. In contrast, *tspC*^*−*^and *tspD*^*−*^ mutants were easily obtained. The genomic integration of the resistance cassette was validated by PCRs (Fig 6B). We performed each of the following experiments with two independent knockout clones, which provided identical results. The *tspC*^*−*^ and *tspD*^*−*^ cells were morphologically intact and grew normally with doubling times of about 10 h in axenic medium. Both mutants maintained the ability to complete a full development cycle within 24 h and to generate viable spores (Fig 7, 24 h). To quantify the motile properties of wildtype and *tspC*^*−*^ or *tspD*^*−*^ *D*. *discoideum* amoebae, we tracked random motility of individual cells in low osmolarity phosphate buffer using time-lapse video recording. The average speed of knockout and wildtype cells was comparable at 7.0 μm min^-1^, respectively 6.8 μm min^-1^ for *tspC*^*−*^and 7.2 μm min^-1^ for *tspD*^*−*^. Chemotaxis towards cAMP seemed equally unaltered, as deduced from developmental assays where *tspC*^*−*^ *and tspD*^*−*^ cells exhibited normal streaming behavior (Figs 7 and 8H). To test for adhesion defects of the deletion strains, wildtype and mutant cells were allowed to adhere to a glass surface and then gently agitated. The non-adhered cells in the supernatant were counted and subtracted from the total cell number as a measure of cell-substrate adhesion. *TspC*^*−*^ *and tspD*^*−*^ cells did not differ from wildtype cells in their ability to adhere to the substratum (89%).

Finally, we tested for osmoregulation defects. Wildtype *D*. *discoideum* cells can cope with severe hypo-osmotic stress by rapidly expelling water through the contraction of vacuoles [37,38]. Wildtype as well as *tspC*^*−*^ and *tspD*^*−*^ cells looked healthy and were amoeboid shaped in standard HL5 media (Fig 8A, 0 min). Then, we challenged the cells by suspending them in distilled water. After initial swelling (Fig 8A, 10 min), wildtype and *tspD*^*−*^ cells regained their normal shape after 20 min (Fig 8A), whereas *tspC*^*−*^ cells were affected more strongly at 10 min and remained round for significantly longer periods of time. Representative images of the cells for the crucial time points can be seen in S2 Fig. However, the cells show a tendency for full recovery after 40 min. For statistical analysis we performed one-way ANOVA with a Bonferroni post-hoc test and found particularly highly significant differences for *tspC*^*−*^ and wildtype cells at 20 and 30 min (Fig 8A, ** = *P* = 0.001) and significant differences for 40 min (Fig 8A, * = *P* < 0.05). To illustrate the *tspC*^*−*^ phenotype, we calculated Δcircularity between 20 and 10 min as a quantitative parameter for initial cell recovery. Fig 8A (right) shows that the initial recovery rate of wildtype (black bar) and *tspD*^*−*^ (white bar) is comparable whereas the value for *tspC*^*−*^ (grey bar) is 2.5x smaller.

In order to analyse the functionality of the contractile vacuoles, living cells were stained with the styryl-dye FM4-64 [37] and followed by confocal microscopy. The dye labelled the plasma membrane immediately and CVs of similar size accumulated in both, wildtype cells and *tspC*^*−*^ mutants (Fig 8B), indicating that their filling efficiency is similar. For quantification, we counted the CVs per cell (Fig 8C). Over half an hour, the number of CVs in the mutant (grey bars) increased at almost double the rate of wildtype cells (black bars). In the time-window from 21 to 30 min, where the circularity of cells diverged most clearly (see Fig 8A), *tspC*^*−*^ cells had significantly more CVs than the wildtype (Fig 8C, * = *P* < 0.05, evaluated with two-tailed student t-test), suggesting that empying of their lumen was less efficient.

To test whether TspC can rescue the *tspC*^*−*^ knockout phenotype, we recombinantly expressed TspC-His (*tspC*^*−*^::TspC-His) in *tspC*^*−*^ cells (*tspC*^*−*^, Fig 8D). Both cell types looked healthy and were amoeboidal-shaped in HL5 media (Fig 8D, 0 min). Suspending the cells in distilled water lead to the anticipated initial swelling (Fig 8B, 10 min). Unlike *tspC*^*−*^ cells, *tspC*^*−*^::TspC-His were able to recover their normal shape after 20 min (student t-test for 20 min ** = *P* < 0.001, Fig 8D). *TspC*^*−*^cells did not even fully recover after 30 min (* = *P* < 0.05, Fig 8D). Two-tailed student t-tests showed no significant difference in circularity for *tspC*^*−*^ and *tspC*^*−*^::TspC-His after 40 min (Fig 8D), reflecting the tendency we saw in the initial osmoregulation experiment (Fig 8A). However, as we also found a not significant but slight increase in circularity for *tspD*^*−*^ cells after 40 min (*P* = 0.282, evaluated with one-way ANOVA with a Bonferroni post-hoc test, Fig 8A), we can not exclude other cell effects that contribute to cell shape at this time point. Calculation of the initial recovery rate shows a 7x increased ability of *tspC*^*−*^::TspC-His to recover after 20 min. This high rate might be explained by overexpression of TspC.

Altogether, these data suggest that TspC plays a role mainly in the initial phases of osmoregulation (10 and 20 min) of *D*. *discoideum* vegetative cells, which is consistent with TspC localization in CVs. However, we currently do not now whether this is really a TspC isoform-specific effect or if each tetraspanin is potentially capable of supporting CV function, if only expressed at a high-enough level.

## Discussion

In most organisms, Tsps usually localize to the plasma membrane where they self-organize in TEMs. Besides, some Tsps are also found in the endosomal system, mainly within late endosomes, lysosmes and in lysosome-related organelles such as α-granules in platelets, melanosomes in melanocytes, cytotoxic granules in T-cells, Weibel–Palade bodies in endothelial cells and Major Histocompatibility Complex II-compartments in dendritic cells [39–42]. In this study, we show that the *D*. *discoideum* vegetative amoebae *TspA*, *TspC* and *TspD* are absent from the plasma membrane and rather co-localize with vacuolar proton pump on CVs. Importantly both, N- and C-terminally GFP tagged Tsps, show the same co-localization with the V-H^+^ ATPase, excluding interference of the GFP with potential N- or C- terminal signaling sequences. In this study, we used endogenous *D*. *discoideum* Tsp sequences for overexpression, ensuring that natural partner proteins as well as post-translational modifications are available. However, it would be interesting to see whether any of the mammalian plasma membrane Tsps would follow a correct trafficking itinerary to the *Dictyostelium* plasma membrane. The phenotype we found for *tspC*^*−*^ is highly consistent with an intracellular localization in the CV, because the *tspC*^*−*^ mutant displayed a clear defect in osmoregulation.

The osmoregulatory organelle in *Dictyostelium* and other free-living amoebae, such as *Trypanosoma*, is the CV network [43,44]. It consists of tubes and bladders. Under hypo-osmotic conditions, as water enters into the cytoplasm, H_3_O^+^ and, most likely driven by antiport HCO_3_^−^ ions are pumped into the CV lumen. Following the osmotic gradient, water streams into the CV, presumably via aquaporins as we have shown previously [45,46]. When the CV reaches its maximal diameter of 2–4 μm, it discharges its content through a pore at the plasma membrane in a giant kiss-and run process [47] and quite a number of proteins have been shown to contribute to this intricate mechanism [48–52]. The precise step in this process, to which TspC contributes, is unknown. However, our data suggest an involvement of TspC in the emptying-phase of CVs (Fig 8C), which appear to be filled normally before and also reach a size similar to wildtype CVs.

Recently it was shown that CVs display functions in addition to coping with hypo-osmotic stress. For instance, CVs facilitate the transport of DdCAD-1, a Ca^2+^ dependent cell-cell adhesion molecule, to the cell surface [53–55]. DdCAD-1 is synthesized as a soluble protein in the cytoplasm and is then transported to the plasma membrane via CVs [53]. From there exocytosis ensues and the protein binds back to the extracellular face of the plasma membrane. Whether one of the Tsp proteins plays a role in this pathway remains to be established. It was also shown that CVs in *Dictyostelium* represent a highly efficient acidic Ca^2+^-store that is required for cAMP induced Ca^2+^ influx [56]. Merely judging from the expression pattern (Table 1), TspB could be a candidate protein involved in this process.

Clearly, more experimental work is necessary to determine the roles that Tsps play in CVs of *D*. *discoideum*. In this context, TspA should be taken into account. Our Western blots (Fig 3B and S1 Fig and Fig 4A) as well as expression time courses from dictyExpress [57] and Fig 2 hint at high expression levels of *TspA* in *D*. *discoideum* amoebae. Until now, we were not able to delete the *TspA* gene by homologous recombination. Further experiments are necessary to address whether the apparent lethality of *tspA*^*−*^ cells can be explained by a requirement for TspA in CV function. Studying phenotypes generated by downregulation of TspA, either by gene knockout or by knockdown via RNAi or by using inducible promotors will be our next attempts to characterize the role of TspA in *D*. *discoideum* vegetative physiology.

Taken together, the characterization and localization of Tsps in *D*. *discoideum* amoebae fills a gap in *Dictyostelium* research, and gives an excellent starting point for further investigations on the functions and protein interactions of Tsps. Although the role of TspC in the CV of *Dictyostelium* may not appear relevant for mammalian cell research, it should be investigated whether similar processes operate in the CVs of pathogenic *Trypanosomes*. In the end, a highly specialized Tsp may one day constitute a valuable drug target for treating sleeping sickness, one of the most threatening tropical diseases.

## Supporting Information

S1 Fig Western blots of GFP-Tsp fusion proteins. Western blots of cell lysates (30 μg per lane) of *D*. *discoideum* vegetative wildtype cells (−) and cells expressing GFP-Tsp using a polyclonal α-GFP antibody as well as the affinity purified TspA antibody. * = unspecific band.
(TIF)

S2 Fig Osmoregulation assay. Bright-field images of *Dictyostelium* AX2 (wt) and *tspC*^*−*^ cells after changing HL5 medium to water at 10, 20 and 30 min. Scale bars = 14 μm.
(TIF)

S1 Table Sequences of PCR primers used in this study. (TIF)
