# Supplementary figures and images for: The C Isoform of Dictyostelium Tetraspanins Localizes to the Contractile Vacuole and Contributes to Resistance against Osmotic Stress
Source: PLoS One. 2016 Sep 6;11(9):e0162065. doi: 10.1371/journal.pone.0162065 (PMC5012570; doi:10.1371/journal.pone.0162065)

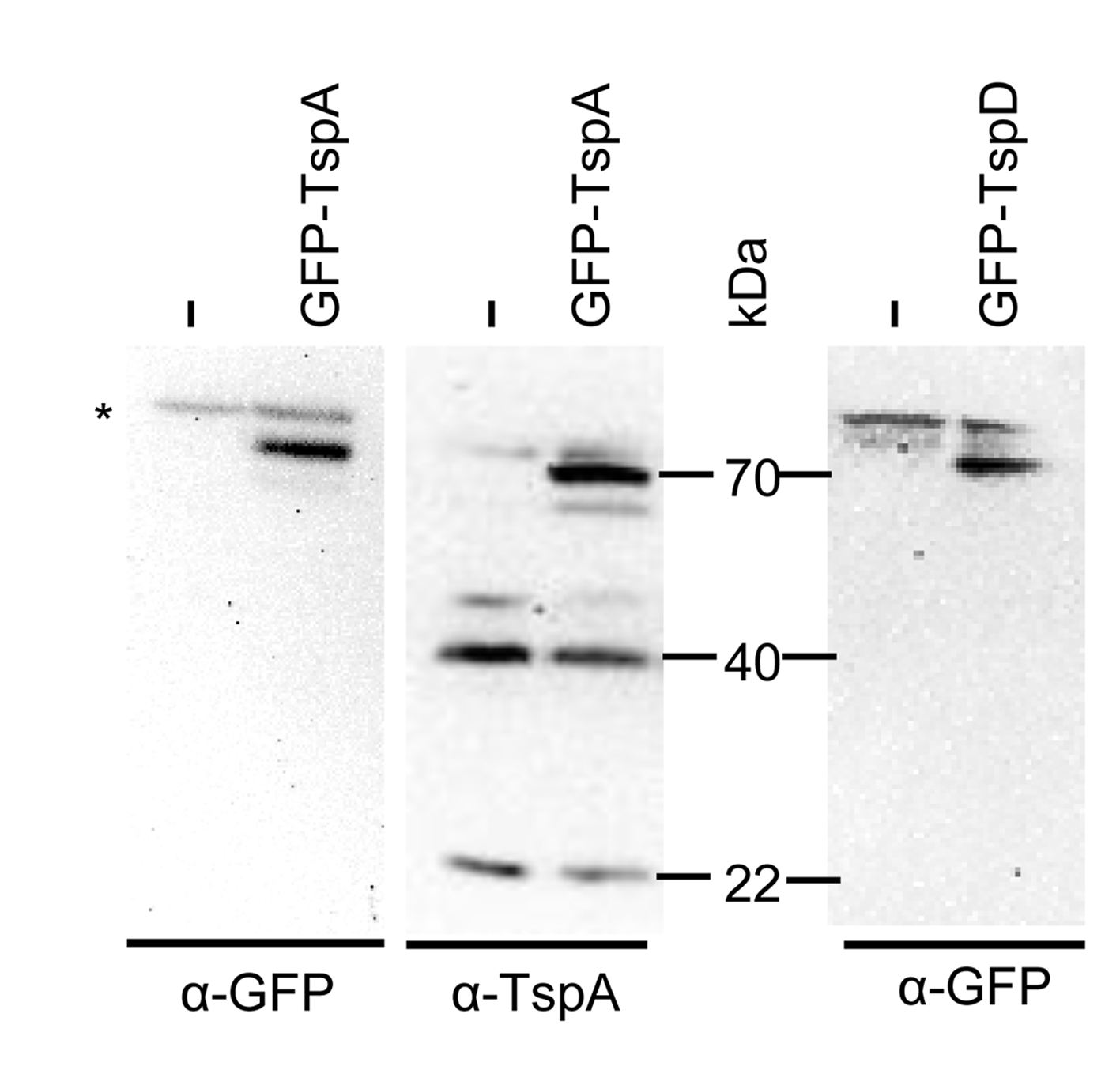

Supplement: S1 Fig — Western blots of cell lysates (30 μg per lane) of D. discoideum vegetative wildtype cells (−) and cells expressing GFP-Tsp using a polyclonal α-GFP antibody as well as the affinity purified TspA antibody. * = unspecific band. (TIF) [file pone.0162065.s001.tif]

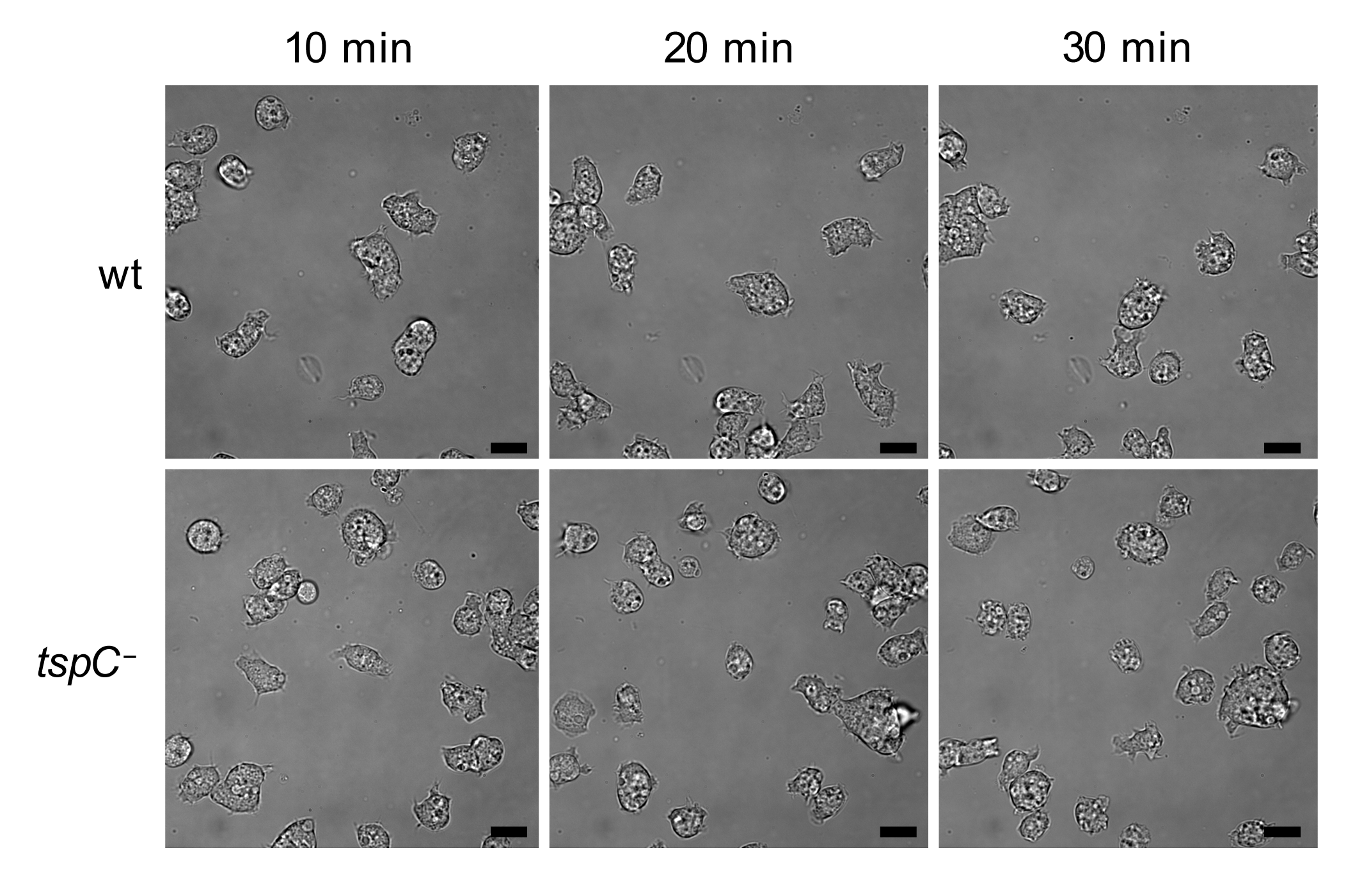

Supplement: S2 Fig — Bright-field images of Dictyostelium AX2 (wt) and tspC− cells after changing HL5 medium to water at 10, 20 and 30 min. Scale bars = 14 μm. (TIF) [file pone.0162065.s002.tif]

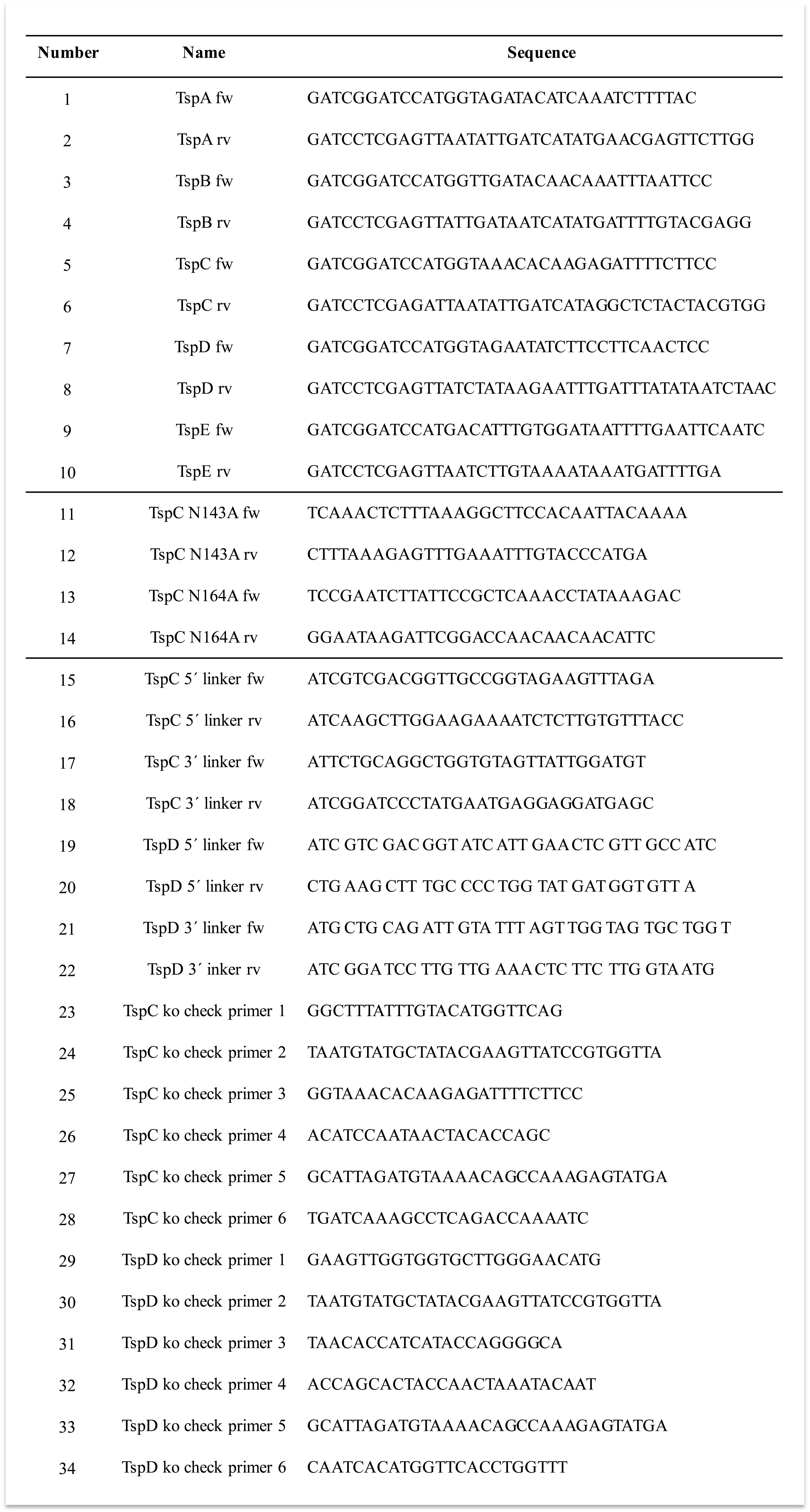

Supplement: S1 Table — (TIF) [file pone.0162065.s003.tif]
